# Calcitonin Peptide Family Members Are Differentially Regulated by LPS and Inhibit Functions of Rat Alveolar NR8383 Macrophages

**DOI:** 10.1371/journal.pone.0163483

**Published:** 2016-10-13

**Authors:** Aichurek Soultanova, Zbigniew Mikulski, Uwe Pfeil, Veronika Grau, Wolfgang Kummer

**Affiliations:** 1 Institute for Anatomy and Cell Biology, Justus Liebig University, Giessen, Germany; 2 Laboratory of Experimental Surgery, Department of General and Thoracic Surgery, Justus Liebig University, Giessen, Germany; 3 Excellence Cluster Cardiopulmonary System (ECCPS), German Center for Lung Research (DZL), Giessen, Germany; University of North Dakota, UNITED STATES

## Abstract

Members of the calcitonin peptide family—calcitonin gene-related peptide (CGRP), adrenomedullin (AM), and adrenomedullin2/intermedin (IMD)–exert modulatory effects upon monocytes and macrophages of various extrapulmonary origins. Utilizing the rat alveolar macrophage (AMφ) cell line NR8383, we here set out to determine to which extent these three peptides and their receptors are differentially regulated in AMφ and what specific effects they have on AMφ key functions. LPS treatment differentially up-regulated expression of the peptides and receptors. Among the three peptides, IMD mRNA content was lowest both in primary rat AMφ and NR8383 cells, whereas IMD peptide dominated in basal and LPS-stimulated secretion from NR8383 cells. Fcγ receptor-mediated phagocytosis and TNF-α production were inhibited by AM, IMD, and CGRP, whereas pro-IL-1β mRNA was slightly down-regulated exclusively by CGRP. Neither of these peptides affected IL-6 or IL-10 production. None increased intracellular calcium concentration, but AM significantly inhibited store-operated calcium entry. In conclusion, the rat AMφ cell line NR8383 is both a source and a target of the calcitonin peptide family members AM, IMD, and CGRP. Despite sharing proteins of the receptor complexes, AM, IMD, and CGRP each showed a characteristic pattern of effects and regulation, suggesting that these closely related peptides are not just redundant members of one common signaling pathway but act in concert by addressing parallel signaling cascades. Since peptide and receptor expression are up-regulated by LPS, these signaling pathways might act as inhibitory feedback mechanisms in pulmonary bacterial infection.

## Introduction

Alveolar macrophages (AMφ) are phagocytes residing on the epithelial lining of airspaces. In normal physiological conditions, they constitute the majority of cells in the bronchoalveolar lavage (BAL) fluid [1]. Equipped with numerous pattern recognition and Fc receptors [2,3], they phagocytose pathogens and particles that have reached alveolar spaces. Activated AMφ are key players in orchestrating pulmonary inflammation by secreting various proinflammatory mediators, including tumor necrosis factor alpha (TNF-α), interleukin 6 (IL-6), IL-1β, IL-8, monocyte chemotactic protein 1 (MCP-1), macrophage inflammatory protein 1α (MIP-1α), proteinases, oxygen radicals, and peroxynitrite [4–8]. Additionally, AMφ play an important role in resolution of inflammation by phagocytosing apoptotic neutrophils and secreting antiinflammatory agents, such as transforming growth factor beta (TGF-β1) and prostaglandin E_2_ (PGE_2_) [9,10]. *In vivo*, depletion of AMφ results in higher mortality and more detrimental lung inflammation during bacterial pneumonia [11,12]. Thus, factors modulating AMφ function may have crucial influence upon maintenance of lung function under physiological and pathophysiological conditions.

There is increasing evidence that the peptides of the calcitonin family, calcitonin gene-related peptide (CGRP), adrenomedullin (AM) and AM2/intermedin (IMD) exert such modulatory effects upon monocytes and macrophages of various origins. CGRP release from peritoneal macrophages is up-regulated by lipopolysaccharide (LPS) [13] and it attenuates release of hydrogen peroxide (H_2_O_2_) and antigen-presenting capabilities of macrophages [14]. AM is beneficial in acute inflammation in that it attenuates acute lung injury [15–17], acute colitis [18,19], acute myocarditis [20], and experimental autoimmune encephalomyelitis [21]. *In vitro*, AM reduces secretion of cytokines by macrophages [22,24] and attenuates hyperpermeability of endothelial cell monolayers [25]. IMD expression is down-regulated in the skin of atopic dermatitis patients [26] and is up-regulated in the testis of LPS-treated rats [27]. Treatment with IMD attenuated orchitis in rats due to decreased production of proinflammatory cytokines and reactive oxygen species [27]. It also decreased foam-cell formation in peritoneal macrophages [28].

These peptides exert their effects via a receptor complex formed by the ligand-binding calcitonin receptor-like receptor (CRLR) and a receptor activity-modifying protein (RAMP) that serves as a chaperone and confers ligand specificity to CRLR [29]. CRLR/RAMP1 acts as a CGRP receptor, CRLR/RAMP2 and CRLR/RAMP3 are AM receptors, whereas IMD binds to all CRLR/RAMP combinations [30]. While studies focusing on either of the peptides have shown that there is plasticity of expression in macrophages *in vitro* or in the whole lung under inflammatory and hypoxic conditions [31–33], it still remains unclear to which extent these three peptides and the receptor proteins are differentially regulated in AMφ, and what specific effects they have on AMφ intracellular Ca^2+^ concentration ([Ca^2+^]_i_), cytokine production and phagocytosis. We here address these questions and show that the rat alveolar macrophage cell line NR8383 is both a source and a target of the calcitonin peptide family members. Peptides and the receptors are up-regulated by LPS in a differential manner. The effects of these peptides exhibit a characteristic pattern suggesting that these closely related peptides are not just redundant members of one common signaling pathway but act in concert by addressing parallel signaling cascades.

## Materials and Methods

### Bronchoalveolar lavage

Eight-week-old female Wistar rats (Charles River, Sulzfeld, Germany) were killed by cervical dislocation under anesthesia with isoflurane (5% in room air). Animals were kept at all times at 12 h light/12 h dark cycle and received food and water *ad libitum*. Animal care and animal experiments were performed following the current version of the German Law on the Protection of Animals as well as the NIH “principles of laboratory animal care” and were registered at the responsible authority (Regierungspraesidium Giessen, JLU 472_M). The trachea was exposed and cannulated with a shortened 14G plastic catheter. The catheter was fixed with a thread and connected to a 10 ml syringe. Five ml of ice-cold phosphate-buffered saline (PBS) without Ca^2+^ (PAA, Pasching, Austria) were injected and aspirated repeatedly until 50 ml of lavage fluid were obtained. The cells were recovered by centrifugation at 400xg, 4°C for 10 min.

### Cell line

The rat AMφ cell line NR8383 (ATCC, catalogue number CRL-2192, LGC Standards, Teddington, Middlesex UK) was cultured at 37°C and 5% CO_2_ in 25 or 75 mm^3^ culture flasks in DMEM/F-12+GlutaMAX medium (Invitrogen, Darmstadt, Germany) supplemented with 15% fetal calf serum (FCS) (Thermo, Langenselbold, Germany), penicillin (100 U/ml) (PAA), and streptomycin (0.1 mg/ml) (PAA). All experiments in this study were repeated at least 4 times on independent days, with N indicating the number of repeated experiments.

### Experimental protocols for mRNA quantification

For analysis of AM, IMD, CGRP, CRLR, and RAMP1-3 gene expression, NR8383 cells were seeded on 6-well plates (5x10^5^ cells per well in 2 ml of the culture medium) and left to adhere for 3 h. The supernatant with unattached cells was discarded and fresh medium with or without LPS (1 μg/ml) (*E*. *coli* serotype O111:B4; Sigma Aldrich, Steinheim, Germany) was added for 3, 6, 12, 24, 48, 72 h. Cells were then detached by incubation with 5 mM ethylenediaminetetraacetic acid (EDTA) (SERVA, Heidelberg, Germany) in PBS (179 mg/l NaH_2_PO_4_x2H_2_O (Merck, Darmstadt, Germany), 685 mg/l Na_2_HPO_4_x2H_2_O (Merck), 4.5 g/l NaCl (Roth, Karlsruhe, Germany) in water, pH = 7.4) for 5 min, and separated from medium by centrifugation at 1000xg for 7 min.

To evaluate effects of AM, IMD, and CGRP on TNF-α mRNA levels, NR8383 cells were seeded on 12-well plates (5x10^5^ cells per well in 0.5 ml of medium) and left to adhere for 3 h. Supernatant with unattached cells was discarded and fresh medium with the following additives was given for 24 h: 1) LPS (100 ng/ml); 2) LPS plus AM (1–100 nM); 3) LPS plus IMD (1–100 nM); 4) LPS plus CGRP (1–100 nM); 5) LPS plus AM plus AM(20–50) (1–100 μM); 6) LPS plus CGRP plus CGRP(8–37) (1–100 μM); 7) LPS plus IMD plus CGRP(8–37) (1–100 μM); 8) LPS plus IMD plus AM(20–50) (1–100 μM) (all peptides from Phoenix Pharmaceuticals, Karlsruhe, Germany). Untreated samples received only fresh medium. Samples treated with LPS alone or with LPS+AM/IMD/CGRP (each of the peptides at 100 nM) were additionally used to quantify pro-IL-1β, IL-6 and IL-10 mRNA levels.

### RNA isolation

For RNA extraction, cells were lyzed with the Mixer Mill MM 301 (Retsch, Haan, Germany) in RLT buffer (RNeasy Mini Kit, Qiagen, Hilden, Germany) supplemented with 1% (v/v) β-mercaptoethanol (Sigma Aldrich). The samples were centrifuged and processed further using RNeasy Mini Kit according to the manufacturer’s instructions. Concentration of isolated RNA was determined using a biophotometer (Eppendorf, Hamburg, Germany).

### cDNA synthesis

For elimination of genomic DNA, 16 μl of RNA (100 μg/ml) were treated with 2 μl of DNase I, Amplification grade (1 U/μl) (Invitrogen) in the presence of 2 μl of 10xDNase I reaction buffer (DNase I kit) for 15 min at 25°C. The digestion was stopped with 2 μl of EDTA solution (25 mM) (DNase I kit) at 65°C for 10 min. For reverse transcription, 2 μl of oligo-dT primers (0.5 μg/μl) (MWG, Ebersberg, Germany), 2 μl of dNTPs (10 mM) (Invitrogen), 4 μl of dithiothreitol (0.1 M) (Superscript II RT kit, Invitrogen), 2 μl of Superscript II reverse transcriptase (RT) (Superscript II RT kit), and 8 μl of reverse transcription buffer (Superscript II RT kit) were mixed with the samples and incubated for 50 min at 42°C, with a subsequent switch to 70°C for 10 min. The reaction was terminated by cooling at 4°C, and cDNA was used immediately or stored at -20°C. As a negative control, RT was omitted from the samples.

### qPCR

**Fig 1:**
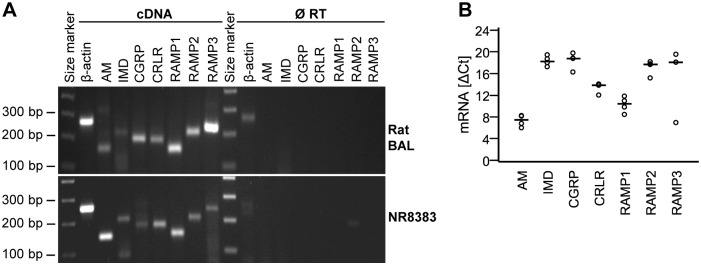
Rat AMφ and NR8383 cells express mRNA for AM, IMD, CGRP, CRLR, and RAMP1-3. A) Agarose gel electrophoresis of RT-qPCR products from cells obtained by bronchoalveolar lavage (BAL) and NR8383 cells. As a negative control, RT was omitted during the cDNA synthesis step (Ø RT). β-Actin served as positive control. Shown is a representative experiment out of 3. B) ΔCt values from RT-qPCR on resting NR8383 cells. Reference gene: β-actin; low values represent high expression. Bar represents median. N = 4.

For quantification of AM, IMD, CGRP, CRLR, and RAMP1-3 gene expression, 2.5 μl of cDNA were mixed with 12.5 μl of SYBR Green Real-Time PCR Supermix (BioRad, Munich, Germany), 0.75 μl of primer mix (forward+reverse, 20 μM each) (MWG; sequences are specified in Table 1), and 9.25 μl of water. For quantification of TNF-α, pro-IL-1β, IL-6, and IL-10 gene expression, 1 μl of cDNA was mixed with 12.5 μl of SYBR Green Real-Time PCR Supermix, 0.75 μl of primer mix, and 10.75 μl of water. The qPCR conditions for AM, IMD, CGRP, and receptors were 5 min at 95°C, 40 cycles of 20 s at 95°C, 20 s at 60°C, and 20 s at 72°C followed by 91 cycles of 10 s at 50°C. Cycling conditions for cytokine quantification were 5 min at 95°C, 40 cycles of 30 s at 95°C, 30 s at 65°C, and 30 s at 72°C followed by 91 cycles of 10 s at 50°C. The Ct values of each target gene were normalized to the Ct values of β-actin, and the relative expression was calculated using the 2^–ΔΔCt^ method. In Fig 1B, the intention was to show expression of the target genes in untreated cells, therefore presentation in ΔCt values was chosen.

**Table 1:** Rat primer sequences.

Target	Forward (5’-3’)	Reverse (5’-3’)	Product length	Gene bank accession no.
β-actin	caaccttcttgcagctcctc	agggtcaggatgcctctctt	252 bp	NM_031144.3
CGRP	gttctcccctttcctggttg	gctccctgactttcatctgc	176 bp	NM_017338.2
AM	gagggaactacaagcgtcca	gaatgtgggctgtgctctga	140 bp	NM_012715.1
IMD	atcagcctcctctacctgct	gcagatgaaccgtagcaagg	199 bp	NM_201426.1
CRLR	tgaaggaaaggttgctgagg	cagagtgggaaaagccattg	173 bp	NM_012717.1
RAMP1	gtggtgtgactggggaaaga	cttgctgaagtagcggtggt	145 bp	NM_031645.1
RAMP2	ctgcggtattgcttggagta	taacgaggaaagggatgagg	199 bp	NM_031646.1
RAMP3	cttctccctctgttgctgct	agcccacgatgtttgtctcc	214 bp	NM_020100.2
TNF-α	tctcattcctgctcgtggcg	ggtgaggagcacgtagtcgg	359 bp	NM_012675.3
IL-6	ttgacagccactgccttccc	cggaactccagaagaccagagc	305 bp	NM_012589.2
pro-IL-1β	ctcgtgctgtctgacccatgt	gctctgcttgagaggtgctga	334 bp	NM_031512.2
IL-10	ccacatgctccgagagctga	tcttcacctgctccactgcc	317 bp	NM_012854.2

### Agarose gel electrophoresis

Amplified cDNA was separated on a gel containing 2% (w/v) agarose (Peqlab, Erlangen, Germany) and 0.001% (v/v) ethidium bromide (Roth) in Tris-acetate-EDTA buffer (TAE, consisting of 4.84 g of TRIS (Roth), 1.142 ml of acetic acid (Merck), 2 ml of 0.5 M EDTA (pH = 8), and water up to 1 L). Electrophoresis was performed in TAE at constant voltage of 150 V for 20–40 min. PCR products were visualized under UV light using a gel documentation system (Phase, Luebeck, Germany).

### Enzyme-linked immunosorbent assay (ELISA)

To analyze secreted AM, IMD, and CGRP, NR8383 cells were seeded on 12-well plates (2x10^6^ cells per well in 0.5 ml of culture medium) and left to adhere for 3 h. The supernatant with unattached cells was removed and fresh medium with or without LPS (1 μg/ml) was added for 1, 6, 24, or 72 h. Culture supernatants were collected, cleared from particulates by centrifugation at 1000xg for 7 min, and stored at -80°C until analysis.

To quantify secreted cytokines, NR8383 cells were seeded on 24-well plates at a density of 10^6^ cells per well in 0.5 ml of culture medium (for analysis of TNF-α and pro-IL-1β) or on 12-well plates at a density of 2x10^6^ cells per well in 0.5 ml of medium (for analysis of IL-6 and IL-10) and left for 3 h to adhere. The culture supernatant with non-adherent cells was discarded and exchanged for fresh medium alone, LPS (100 ng/ml), AM (100 nM), IMD (100 nM), CGRP (100 nM), or combinations of LPS and one of the peptides. After 24 h of stimulation, culture medium was collected, centrifuged at 1000xg for 7 min, and stored at -80°C until analysis. ELISA measurements were performed according to the manufacturers’ instructions (kits for AM, IMD and CGRP: Phoenix Pharmaceuticals; kits for cytokines: Affymetrix ebioscience, Frankfurt, Germany). Optical density (OD) was read at 450 nm using ELISA plate reader; OD at 570 nm was subtracted as background. Concentrations of samples were interpolated from the standard curve.

### Phagocytosis assay

NR8383 cells were seeded on 8-well culture slides (BD Falcon, Heidelberg, Germany) at a density of 2x10^4^ cells per well in 300 μl of culture medium and were allowed to adhere overnight. Unattached cells were removed and the remaining cells were pretreated with ATP (100 μM) (Sigma Aldrich), PGE_2_ (1 μM) (Sigma Aldrich), AM (1 μM), IMD (1 μM), or CGRP (1 μM) in fresh medium for 15 min at 37°C and 5% CO_2_. Phagocytosis was stimulated by incubation for 30 min with opsonized Texas Red-conjugated zymosan A *S*. *cerevisiae* beads (Invitrogen) at a ratio of 40 beads per cell with or without substances of interest. The beads were opsonized by incubation for 1 h with rabbit anti-zymosan antiserum (Invitrogen) at a ratio of 1:1 followed by 3 washings by centrifugation at 1000xg for 15 min. Phagocytosis was stopped by placing the culture slides on ice, cells were washed 4 times with ice-cold PBS and fixed in 4% paraformaldehyde (Merck) for 10 min. Non-specific binding sites were saturated by incubation with a mixture of 5% bovine serum albumin (Sigma Aldrich) and 5% FCS in PBS. Cell membranes were stained with biotinylated IB4 (2 μg/ml, 1 h) (Sigma Aldrich) and streptavidin-fluorescein isothiocyanate (FITC) (2 μg/ml, 20 min) (Sigma Aldrich), cell nuclei were stained with 4',6-diamidino-2-phenylindole (DAPI) (0.2 μg/ml, 5 min) (Sigma Aldrich). All incubations were performed at room temperature. Slides were examined with a fluorescence microscope (Axioplan 2, Zeiss, Goettingen, Germany), and photographs were taken of 10 randomly chosen fields of view while visualizing cells by aid of DAPI fluorescence. The total number of beads and cells on each photograph were counted and the phagocytosis index (PI = number of engulfed beads per cell) was determined.

### Calcium measurements

NR8383 cells were seeded on 12 mm (Ø) coverslips (Roth) at a density of 3x10^4^ cells per coverslip in 80 μl of medium and were allowed to adhere for 20 min. Coverslips were washed twice with warm HEPES buffer (5.6 mM KCl (Merck), 136.4 mM NaCl, 11 mM D(+)Glucose (Sigma Aldrich), 10 mM HEPES (Roth), 1 mM MgCl_2_ (Merck), 2.2 mM CaCl_2_ (Merck) in water, pH = 7.4) and incubated with Fura-2AM (3.3 ng/μl) (Invitrogen) for 30 min at 37°C. After 30 min, coverslips were washed three times in HEPES buffer and loaded on a 0.17 mm-thick delta T dish (Bioptech, Butler, USA). At 40^th^, 100^th^, and 160^th^ s of the imaging, AM, IMD, CGRP (each at 10 nM, 100 nM, and 1 μM), acetonitrile (Fluka Chemika, Buchs, Switzerland), or HEPES buffer (the latter two are vehicle controls) were added to the delta T dish. At the 220^th^ s, ATP (100 μM) was applied as a control of the cells’ reactivity.

For analysis of SOCE, cells were kept in Ca^2+^-free HEPES buffer (5.6 mM KCl, 136.4 mM NaCl, 11 mM D(+)Glucose, 10 mM HEPES, 1 mM MgCl_2_ in water, pH = 7.4) at the beginning of the recording, and thapsigargin (100 μM) (Sigma Aldrich) was applied at the 20^th^ s to deplete intracellular Ca^2+^ stores. AM, IMD, or CGRP (each at 100 nM) were applied at the 300^th^ s, and SOCE was triggered at the 420^th^ s by exchanging Ca^2+^-free HEPES buffer for Ca^2+^-containing HEPES buffer.

Recordings of [Ca^2+^]_i_ were done using a fluorescence microscope (Olympus, Hamburg, Germany) connected to a scan CCD camera with fast monochromator (TiLL Photonics, Gräfelfing, Germany). Fura-2AM was excited at 340 and 380 nm, and emission intensity was measured at 420 nm. For each recorded cell, the ratio 340/380 nm was obtained. Ratio values at each second of the experiment were normalized to the values of the 1^st^ s.

### Statistics

**Fig 2:**
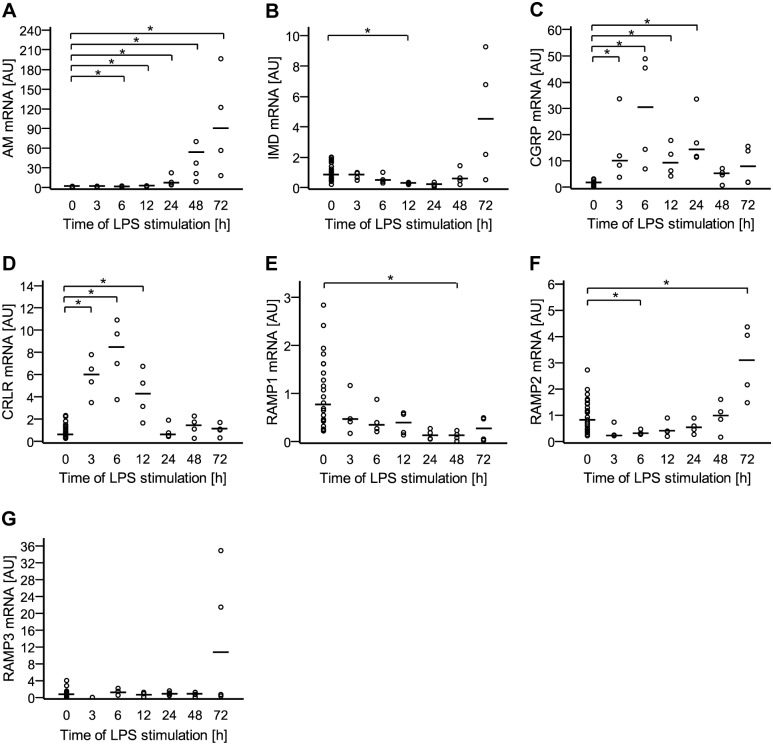
Messenger RNA content of peptides and their receptor complex is regulated by LPS. NR8383 cells were treated with LPS (1 μg/ml) or left untreated. At indicated time points, gene expression of A) AM, B) IMD, C) CGRP, D) CRLR, E) RAMP1, F) RAMP2, and G) RAMP3 was analyzed by RT-qPCR. Data is presented in arbitrary units (AU) of mRNA expression with untreated cells set as 1. Bar represents median. N = 4 (N = 24 for untreated) *, p≤0.05 (Mann-Whitney test). P value for IMD at 72 h = 0.2 compared to untreated; p value for RAMP3 at 72 h = 0.057 compared to untreated.

For statistical analysis, SPSS Statistics software (IBM, Munich, Germany) was employed. Statistical significance between multiple groups (K>2) was analysed by nonparametric rank-sum Kruskal-Wallis test. In case of significance (p≤0.05), nonparametric rank-sum Mann-Whitney test was used to compare between individual groups. Power analysis of the data set depicted in Fig 2 was provided by Anita Windhorst, Institute for Medical Informatics, JLU Giessen.

## Results

### Rat AMφ and NR8383 cells express AM, IMD, CGRP, CRLR, and RAMP1-3

Expression of the peptides and their receptors was analyzed using RT-qPCR. Both rat AMφ and NR8383 cells express mRNA for AM, IMD, CGRP, CRLR, RAMP1, RAMP2, and RAMP3 (Fig 1A). Quantitative analysis of NR8383 cells showed the following abundance of the mRNA expression: AM >IMD = CGRP (Fig 1B).

### Messenger RNA expression of peptides and their receptor complex in NR8383 cells is regulated by LPS

Exposure to LPS up-regulated AM and CGRP mRNA in a time-dependent manner (Fig 2A and 2C). AM expression increased gradually, starting at 6 h of stimulation (1.6-fold) and reached ~100-fold increase at 72 h (Fig 2A). CGRP mRNA, in contrast, had the highest expression at 6 h (30-fold) and declined to the initial values afterwards (Fig 2C). IMD mRNA was down-regulated by LPS treatment (0.27-fold) at 12 h, and then returned to initial values. At 72 h, the median was about 5-fold higher than baseline, but the data did not differ significantly from baseline (Fig 2B). It has to be noted, however, that in this particular data set a power of only 57% was calculated. The time curve of CRLR up-regulation was similar to CGRP (Fig 2D), RAMP1 was down-regulated at 48 h (Fig 2E), RAMP2 was initially down-regulated and then up-regulated (Fig 2F), and RAMP3 expression did not change significantly. Similar to IMD, a much higher median value of RAMP3 was observed at 72 h, but again, a small statistical power (47%; for comparison: 83% and 87% for AM 72 h and RAMP2 72 h data, respectively) was calculated (Fig 2G).

### AM, IMD, and CGRP are secreted by NR8383 cells, and their release is enhanced by LPS

**Fig 3:**
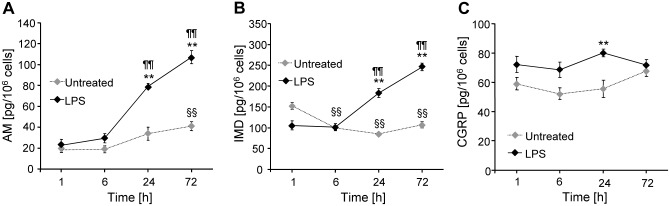
LPS enhances secretion of AM, IMD, and CGRP by NR8383 cells. NR8383 cells were incubated in fresh culture medium with or without LPS (1 μg/ml). At indicated time points, the concentration of A) AM, B) IMD, and C) CGRP in the media was measured using colorimetric competitive ELISA. Data is presented as mean±SEM (N = 5). **, p≤0.01, compared to untreated; §§, p≤0.01, compared to untreated at 1 h; ¶¶, p≤0.01, compared to LPS at 1 h (Mann-Whitney test). Raw data are available as S1 File.

Competitive ELISA showed that NR8383 cells secreted AM, IMD, and CGRP under unstimulated conditions, and secretion was significantly enhanced after 24 h of treatment with LPS (Fig 3). After 72 h of stimulation, AM and IMD release was further increased (Fig 3A and 3B), whereas CGRP levels did not differ between the groups (Fig 3C).

### AM, IMD, and CGRP do not directly affect [Ca^2+^]_i_, but AM inhibits SOCE

**Fig 4:**
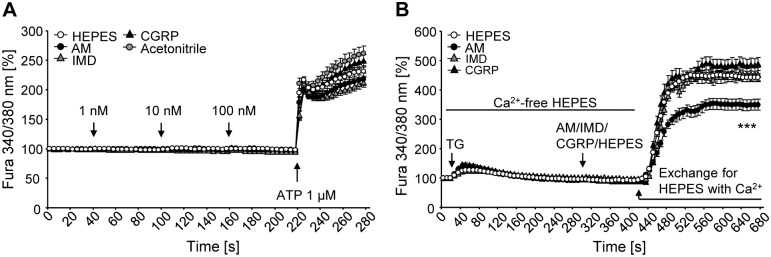
AM, IMD, and CGRP do not have direct effect on [Ca^2+^]_i,_ but AM inhibits SOCE. Ratiometric measurements of [Ca^2+^]_i_. A) N = 190 (HEPES buffer), 106 (AM), 124 (IMD), 118 (CGRP), and 98 cells (acetonitrile, vehicle control). B) To analyse SOCE, cells were kept in Ca^2+^-free HEPES buffer, then thapsigargin (TG) (100 μM) was applied, followed by peptides. N = 153 (HEPES buffer), 84 (AM), 42 (IMD), and 46 cells (CGRP). Data is presented as ratio of fluorescence after excitation at 340/380 nm normalized to the 1^st^ s. ***, p≤0.001 (AM versus HEPES buffer; Mann-Whitney test). Raw data are available as S2 File.

We tested whether AM, IMD, and CGRP modulate intracellular Ca^2+^ levels. Successive application of increasing concentrations of AM, IMD, or CGRP had no direct effect on [Ca^2+^]_i_ (Fig 4A). Subsequent addition of ATP resulted in a drastic increase in [Ca^2+^]_i_, which was unaffected by previous treatments with peptides (Fig 4A).

To study effects of AM, IMD, and CGRP on SOCE, cells were kept in Ca^2+^-free HEPES buffer immediately before the experiment and during the first part of it. Intracellular calcium stores were depleted by addition of thapsigargin. It induced a slight increase in [Ca^2+^]_i_, which then turned into a slow decline until reaching basal values (Fig 4B). Application of AM, IMD, or CGRP had no direct impact on [Ca^2+^]_i_. Exchange of Ca^2+^-free HEPES buffer to Ca^2+^-containing HEPES buffer triggered a very pronounced rise in [Ca^2+^]_i_, which after reaching its maximum value at about 100 s, stayed constant until the end of the readings (Fig 4B). This influx of Ca^2+^ from the extracellular space was not different between IMD-, CGRP-, and HEPES buffer-treated cells. AM, in contrast, inhibited SOCE by 25% (Fig 4B).

### AM, IMD, and CGRP inhibit phagocytosis

**Fig 5:**
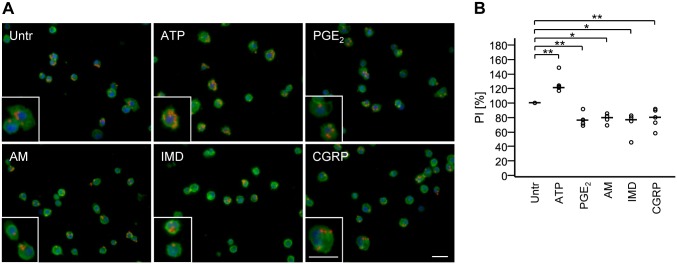
AM, IMD, and CGRP inhibit phagocytosis. A) NR8383 cells, treated with AM (1 μM), IMD (1 μM), CGRP (1 μM), PGE_2_ (1 μM), or ATP (100 μM) were exposed to opsonized Texas Red-conjugated zymosan beads for 30 min. Nuclei were stained with DAPI (blue), cell membranes were stained with FITC-labeled IB4 (green). ATP is a control of positive regulation; PGE_2_ is a control of negative regulation. Shown are representative pictures from 6 experiments. Bar, 50 μm. B) Phagocytosis activity is presented as phagocytosis index, normalized to untreated. Bar represents median. N = 5 experiments (N = 4 for AM and IMD). *, p≤0.05, **, p≤0.01 (Mann-Whitney test).

To evaluate impact of AM, IMD, and CGRP on Fcγ receptor-mediated phagocytosis, NR83833 cells were stimulated with opsonized Texas Red-conjugated zymosan beads (Fig 5A). Treatment with ATP increased phagocytosis, whereas PGE_2_, AM, IMD, and CGRP attenuated it (Fig 5B).

### AM, IMD, and CGRP attenuate production of TNF-α

**Fig 6:**
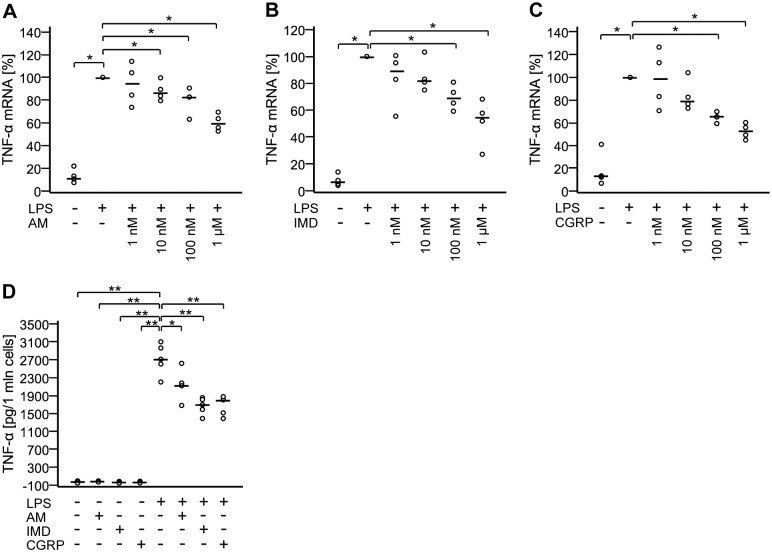
AM, IMD, and CGRP attenuate production of TNF-α. A-C) NR8383 cells were treated with LPS (100 ng/ml) with or without AM, IMD, or CGRP at indicated concentrations for 24 h. TNF-α mRNA expression was analyzed by RT-qPCR. Data is presented as relative mRNA expression, normalized to LPS-treated cells. Bar represents median. N = 4. *, p≤0.05 (Mann-Whitney test). D) NR8383 cells were treated with LPS (100 ng/ml) with or without AM, IMD, or CGRP (each at 100 nM) for 24 h and TNF-α levels in the culture media were measured by ELISA. Bar represents median. N = 5. *, p≤0.05; **, p≤0.01 (Mann-Whitney test).

Effects of AM, IMD, and CGRP on LPS-induced TNF-α production were analyzed using RT-qPCR and ELISA. TNF-α mRNA expression was down-regulated dose-dependently upon treatment with AM, IMD, or CGRP (EC_50_ values are 125 nM, 114 nM, and 44 nM, respectively) (Fig 6A–6C). Consistently, levels of secreted TNF-α were also reduced after administration of either of the peptides (Fig 6D).

**Fig 7:**
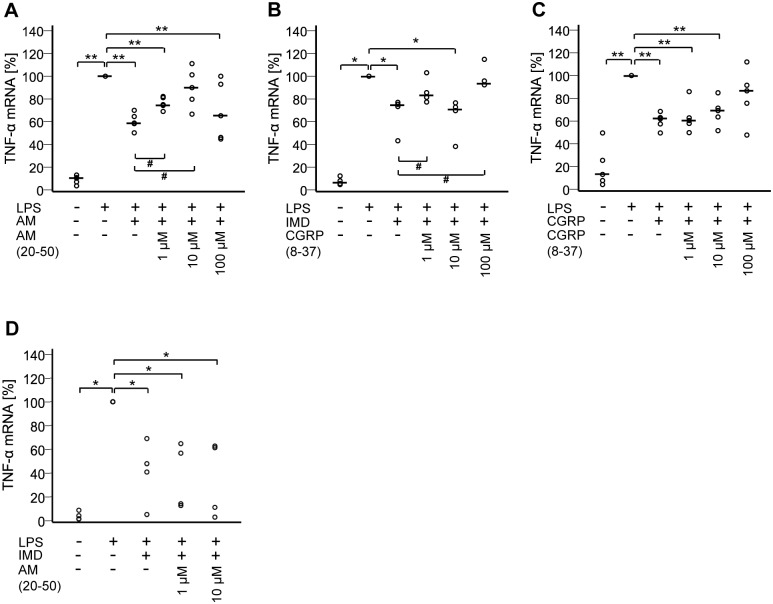
Effects of AM, IMD, and CGRP on TNF-α mRNA are receptor-mediated. NR8383 cells were treated for 24 h with LPS (100 ng/ml) with or without A) AM (100 nM) and indicated concentrations of AM(20–50); B) IMD (100 nM) and indicated concentrations of CGRP(8–37); C) CGRP (100 nM) and indicated concentrations of CGRP(8–37); D) IMD (100 nM) and indicated concentrations of AM(20–50). TNF-α mRNA expression was analyzed by RT-qPCR. Data is presented as relative mRNA expression, normalized to LPS-treated cells. Bar represents median. N = 5 (N = 4 for IMD). *, p≤0.05; **, p≤0.01; #, p≤0.05 (Mann-Whitney test).

To test whether effects of AM, IMD, and CGRP on TNF-α transcription are receptor-mediated, the antagonistic peptides AM(20–50) and CGRP(8–37) were used. AM(20–50) is a blocker of complexes CRLR/RAMP2 and CRLR/RAMP3, whereas CGRP(8–37) is a blocker of the CRLR/RAMP1 complex. As demonstrated in Fig 7A, AM(20–50) at 1 μM and 10 μM attenuated the inhibitory effect of AM (p≤0.05 compared to LPS+AM; 10 μM was not significantly different from LPS). Effects of IMD were decreased by CGRP(8–37) at 1 μM and 100 μM (p≤0.05 compared to LPS+IMD and not significantly different compared to LPS), but not by AM(20–50) (Fig 7B and 7D). Effects of CGRP were attenuated by 100 μM CGRP(8–37) (at this concentration the response was no longer significantly different from LPS) but not at lower antagonist concentrations (Fig 7C).

### CGRP decreases LPS-induced pro-IL-1β mRNA levels, but not release of IL-1β

**Fig 8:**
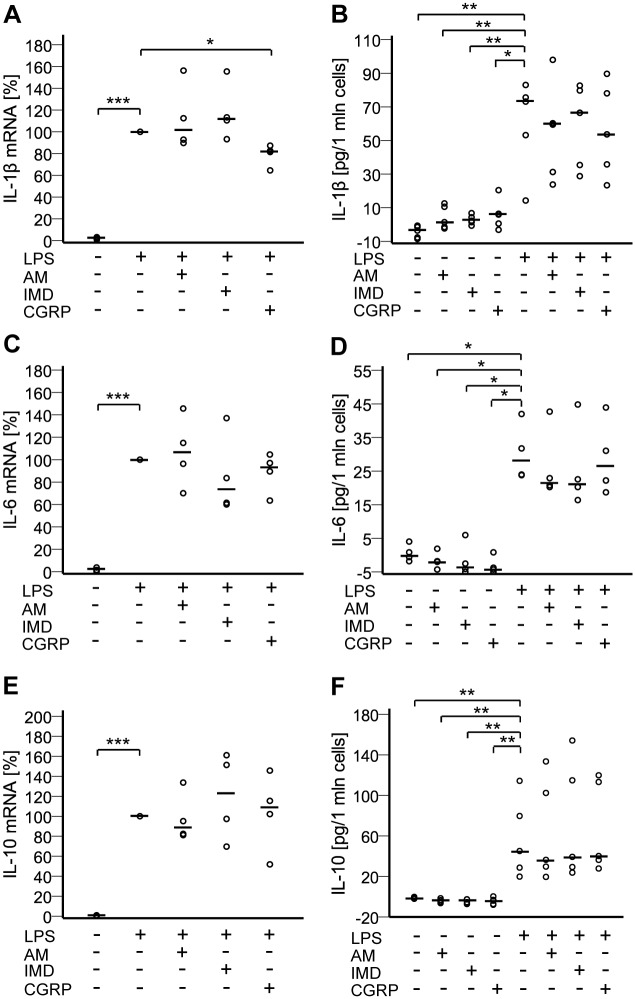
CGRP decreases LPS-induced IL-1β mRNA levels. NR8383 cells were treated for 24 h with LPS (100 ng/ml) with or without AM, IMD, or CGRP (each at 100 nM). A), C), E) Messenger RNA expression of pro-IL-1β, IL-6, and IL-10 was measured by RT-qPCR. Data is presented as relative mRNA expression, normalized to LPS-treated cells. Bar represents median. N = 4 (N = 12 for untreated). *, p≤0.05; ***, p≤0.001 (Mann-Whitney test). B), D), F) Levels of IL-1β (N = 5), IL-6 (N = 4), and IL-10 (N = 5) in the culture media were measured by ELISA. Bar represents median. *, p≤0.05; **, p≤0.01 (Mann-Whitney test).

Classical protocols for stimulation of IL-1β secretion use a first stimulus (e.g. LPS), which induces pro-IL-1β synthesis and accumulation in cytosol, and a second stimulus (P2X7 receptor agonist), which leads to cleavage and release of IL-1β [34]. Since NR8383 cells lack P2X7 receptors [35], we used a single stimulation with LPS. LPS up-regulated expression of pro-IL-1β mRNA, which was significantly reduced in the presence of CGRP (Fig 8A). AM and IMD did not change pro-IL-1β mRNA amounts (Fig 8A). IL-1β secretion was affected by neither of the peptides (Fig 8B). Messenger-RNA content of IL-6 and IL-10 was up-regulated by LPS treatment (Fig 8C and 8E). This increase was unaffected by addition of AM, IMD, or CGRP (Fig 8C and 8E). At the protein level, unstimulated cells did not secrete detectable IL-6 or IL-10, whereas LPS induced their release. Neither of the peptides exerted any effects on the levels of secreted cytokines (Fig 8D and 8F).

## Discussion

The present data demonstrates that rat AMφ/NR8383 cells are both a source and a target of the calcitonin peptide family members, AM, IMD, and CGRP. Among these three, IMD mRNA content was lowest both in rat AMφ and NR8383 cells, whereas IMD peptide dominated in basal, constitutive secretion from NR83838 cells, reaching more than 7 times higher levels compared to AM and 2.5 times higher levels compared to CGRP. This might be explained by IMD mRNA instability, as it is known also from the inflammatory mediators TNF-α, pro-IL-1β, GM-CSF, and interferons [36]. In light of these findings, caution must be taken when interpreting tissue abundance of IMD based solely on mRNA data.

Treatment with LPS regulated both mRNA content and peptide secretion of all three peptides, albeit each in a specific manner and time course. While AM mRNA showed a late response, with a maximum up-regulation seen at 72 h of stimulation, CGRP mRNA increase was rapid, with a peak at 6 h and subsequent return to basal values. This transient time course is consistent with a report from Ma and colleagues, showing maximally enhanced release of CGRP from RAW264.7 macrophages after 24 h of LPS stimulation and a decline thereafter [33]. IMD mRNA, on the opposite, was temporarily slightly down-regulated by LPS. On a relative basis, AM was most prominently regulated with an approximately 100-fold increase in mRNA and 5-fold increase in secreted peptide 72 h after LPS treatment, resulting in a switch in the rank order of peptide abundance in the medium from IMD>CGRP>AM before to IMD>AM>CGRP after stimulation. Components of the receptor complexes were also differentially regulated, in that LPS up-regulated CRLR and RAMP2 expression, but not RAMP1 and RAMP3. Collectively, these data show an overall up-regulation of the calcitonin peptide family signaling system in NR8383 cells by LPS, pointing towards a potential modulatory role in inflammation.

Despite at least partial convergence at the receptor level—CRLR/RAMP1 is addressed by both CGRP and IMD, and CRLR/RAMP2/3 by both AM and IMD—all three peptides had an individual pattern of inhibitory effects upon NR8383 cell functions. So far, comparative data on the effects of these peptides on phagocyte functions have only been provided for mouse osteoclasts and their progenitor cells, also showing a differential effector pattern. There, IMD and CGRP, but not AM, inhibited bone resorption in mouse calvarial bones and decreased formation of osteoclasts, although all three stimulated cAMP formation in macrophage-colony stimulating factor-expanded osteoclast progenitor cells lacking calcitonin receptors [37]. This was interpreted to be due either to downstream inhibition of AM signaling or to the presence of unknown AM receptors distinct from CRLR/RAMPs [37]. In order to test for effects unrelated to CRLR/RAMPs, we used the receptor antagonists CGRP(8–37), which targets CRLR/RAMP1 complex, and AM(20–50), which blocks CRLR/RAMP2 and CRLR/RAMP3 complexes. Earlier studies showed that CGRP(8–37) antagonizes the majority of CGRP effects, but has much weaker antagonistic potency in the rat vas deferens [38], suggesting that CGRP may act additionally on AM or amylin receptors. In our experiments, the AM effects on LPS-induced TNF-α mRNA up-regulation were sensitive to AM(20–50) at 1 μM concentration which is well compatible with being mediated by CLRL/RAMP2/3 complexes. Still, the EC_50_ values of AM, IMD and CGRP and the CGRP(8–37) concentrations needed to antagonize the down-regulatory effect of CGRP on LPS-induced TNF-α mRNA up-regulation were higher than reported for other systems [39–42]. This might be caused by breakdown of peptide agonists and antagonists by peptidases or unspecific mechanisms during the 24 h period of the experimental protocol. Alternatively, there are more complex signaling pathways underlying the observed effects which do not entirely rely on CRLR/RAMPs, as it had also been suggested for bone remodeling [37].

In addition to the canonical signaling pathway of AM, IMD, and CGRP involving activation of G_s_α and increase of intracellular cAMP levels [43], another signal transduction pathway engaging calcium ions as secondary messenger has also been proposed [44,45]. We addressed this notion using ratiometric measurements of [Ca^2+^]_i_. AM, IMD, and CGRP had no direct effect on intracellular calcium levels, thereby not supporting the notion of activation of a G_αq/11_-dependent pathway via CRLR. Still, AM, but neither IMD nor CGRP, interfered with regulation of [Ca^2+^]_i_ in NR8383 cells. Non-excitable cells replenish their intracellular calcium depot via SOCE. Upon emptying of the endoplasmic reticulum, a signal is sent to store-operated channels in the plasma membrane, which open and enable Ca^2+^ influx [46]. AM, but neither IMD nor CGRP, significantly inhibited SOCE in NR8383 cells. Thus, signaling pathways of AM, despite shared receptors, are at least in part different from IMD and CGRP, as it had been also proposed for mouse osteoclasts [37].

Several previous reports showed that AM, IMD, and CGRP attenuate production of proinflammatory cytokines in the setting of inflammation [22,23,27,47]. Accordingly, we noted a clear inhibitory effect of all of these peptides on LPS-induced increase in TNF-α mRNA and peptide secretion in NR8383 cells. However, except for a slight decrease in IL-1β mRNA upon CGRP treatment, we did not observe effects of these peptides on production of IL-6, IL-1β and IL-10. This contradicts previous reports where AM, CGRP, and IMD modulated not only TNF-α, but also IL-1β and IL-6 expression and secretion in gastrointestinal tract, testis, microglia, and THP-1 macrophage-like cells [27,48–50]. Moreover, Wong et al., using the same cell line and the same concentrations of LPS and AM, demonstrated potentiation of LPS-induced up-regulation of IL-6 upon AM treatment at 3 h and 6 h of incubation [22]. These early time points were not included in our measurements, which were conducted after 24 h.

Effective phagocytosis of inhaled particles is a hallmark function of AMφ, triggering subsequent production of proinflammatory cytokines [51]. It is subject to modulation by intracellular cAMP levels with high cAMP levels inhibiting, and low cAMP levels enhancing phagocytosis [52–54]. In contrast to the differential and modest impact upon cytokine production, AM, IMD, and CGRP all effectively attenuated phagocytosis of opsonized zymosan beads by NR8383 cells to the same extent as PGE_2_, a potent and well established inhibitor of phagocytosis acting via EP2 receptors and activation of adenylate cyclase [52]. In response to phagocytosis of apoptotic cells, AMφ release PGE_2_, which then acts as an autoinhibitory feedback signal to limit further phagocytosis and proinflammatory cytokine production [9,55]. In view of the presently demonstrated LPS-induced up-regulation of AM, IMD, and RAMP2 in NR8383 cells, this signaling pathway may function in a similar manner as an auto-/paracrine inhibitory feedback mechanism in bacterial infection.

In conclusion, the rat AMφ cell line NR8383 is both a source and a target of the calcitonin peptide family members, AM, IMD, and CGRP. Most prominent effects seen in this study are inhibition of phagocytosis and TNF-α production, and since peptide and receptor expression are up-regulated by LPS, these signaling pathways might act as inhibitory feedback mechanisms in bacterial infection. Despite sharing components of the receptor complexes, AM, IMD, and CGRP each showed a characteristic pattern of effects and regulation, suggesting that these closely related peptides are not just redundant members of one common signaling pathway but act in concert by addressing parallel signaling cascades.

## Supporting Information

S1 File ELISA raw data and calculations. (XLS)

S2 File Raw data of [Ca^2+^]_i_ measurements. (XLS)
